# Commentary: A GSH/CB dual-controlled self-assembled nanomedicine for high-efficacy doxorubicin-resistant breast cancer therapy

**DOI:** 10.3389/fphar.2022.1081912

**Published:** 2022-11-28

**Authors:** Tian Tian

**Affiliations:** Department of Neurology, The First Affiliated Hospital of Zhengzhou University, Zhengzhou, China

**Keywords:** breast cancer, cell death, apoptosis, chemoresisitance, exon deletion

## Introduction

With great interest, we read the paper *A GSH/CB dual-controlled self-assembled nanomedicine for high-efficacy doxorubicin-resistant breast cancer therapy* published in the Frontiers in Pharmacology (Yang et al., 2021). In the study, Yang et *al* developed a glutathione (GSH)/cathepsin B (CB) dual-controlled precursor of a cyclic disulfide-bridged peptide (cyclic-1a). It could form a supramolecular hydrogel to overcome the drug resistance in breast cancer. Overall, this is an excellent study, but some issues need to be discussed here.

In Yang’s report, the human breast cancer MCF7 cells were used to determine the anticancer efficacy of cyclic-1a. The results showed that various concentrations of cyclic-1a promoted the apoptosis of MCF-7 doxorubicin resistant cells in a dose-dependent manner. Moreover, the total caspase-3 protein markedly down-regulated, while cleaved caspase-3 protein up-regulated significantly after treatment with cyclic-1a (Figure 4C and Figure 5E).

However, it is well known that the MCF-7 cells do not express caspase-3 (Jänicke et al., 1998; Friedrich et al., 2001; Ferguson et al., 2003; Jänicke, 2009). The lack of caspase-3 in MCF-7 cells is caused by a forty-seven base pair deletion within exon 3 of the *CASP-3* gene. It leads to skip the exon during pre-mRNA splicing and introduce a premature stop codon, which abrogates translation of the *CASP-3* mRNA completely (Jänicke et al., 1998; Friedrich et al., 2001; Ferguson et al., 2003; Jänicke, 2009). Therefore, the detection of the caspase-3 protein in caspase-3 deficient MCF-7 cells in this study made us confused.

## Discussion

Such discrepant results might be partially explained by the use of inappropriate antibodies that cross-react with other unrelated proteins of caspase-3 in MCF-7 cell extracts (Jänicke, 2009). Alternatively, the examined cultures might not be the original human breast cancer MCF-7 cells, and the identity of MCF-7 cells needs to be confirmed by short tandem repeat (STR) profiling or any other methods.

In conclusion, we appreciate the authors’ efforts in investigating the effects of cyclic-1a to overcome drug resistance in breast cancer. Nevertheless, we sincerely suggest that appropriate modification may further solidify the findings of the study.
